# Effect of Polypropylene and Straw Fiber Materials on the Unconfined Compressive Strength of Tailings and Wasted Stone Mixed Backfill

**DOI:** 10.3390/ma18020392

**Published:** 2025-01-16

**Authors:** Xiuzhi Shi, Yuan Shi, Xin Chen, Wenyang Wang

**Affiliations:** 1 School of Resources and Safety Engineering, Central South University, Changsha 410083, China; baopo@csu.edu.cn (X.S.); shiyuan@csu.edu.cn (Y.S.); wenyang.wang@csu.edu.cn (W.W.); 2 Sinosteel Maanshan General Institute of Mining Research Co., Ltd., Maanshan 243000, China; 3 School of Civil and Resources Engineering, University of Science and Technology Beijing, Beijing 100083, China; 4 Fankou Lead-Zinc Mine, Shaoguan 512325, China

**Keywords:** polypropylene fiber, straw fiber, tailings, unconfined compressive strength, wasted stone

## Abstract

Ensuring the mechanical performance of backfill materials while reducing cementation costs is a key challenge in mine backfill research. To address this, fiber materials such as polypropylene (PP) fiber and rice straw (RS) fiber have been incorporated into cement-based mixtures for mine backfilling. This study investigates the effects of PP and RS fibers on the mechanical properties, flow characteristics, and microstructure of Tailings and Wasted Stone Mixed Backfill (TWSMB). A series of orthogonal experiments were designed to evaluate the influence of variables, including the cement–sand ratio, solid mass concentration, wasted stone mass concentration, fiber content, and fiber length on the TWSMB properties. The results indicate that the influence of cement–sand ratio and solid mass concentration have a more significant impact on strength than fibers, though the fibers show a stronger effect than the wasted stone mass concentration. Both fiber types enhanced the strength of the specimens, with PP fiber exhibiting a stronger reinforcing effect than RS fiber. Furthermore, the effect of PP fiber content was more pronounced than that of fiber length, whereas the opposite trend was observed for RS fiber. The optimum fiber parameter levels were determined for each type: PP fiber performed best at a mass concentration of 1.5% and a length of 6 mm, while RS fiber showed optimal performance at a mass concentration of 1.0% and a length of 5–10 mm. Macroscopic damage analysis indicated that the structural integrity and residual compressive strength of the TWSMB specimens were preserved even after surpassing the ultimate compressive strength, due to the crack-bridging effect of the fibers. Microstructural analysis showed that PP fiber-reinforced specimens exhibited a dense structure formed through reactions with other hydration products. In contrast, the surface of RS fibers was nearly fully encapsulated by hydration products, resulting in the formation of a physical skeleton structure. This study provides new insights into minimizing cement consumption and reducing backfilling costs in mining operations.

## 1. Introduction

In China, with growing public awareness of environmental protection and safety, domestic underground mines are increasingly focusing on backfilling of mined-out areas [1,2,3]. The cemented paste filling has emerged as an effective and widely adopted backfill method in mining; however, the use of cement leads to increased CO_2_ emissions, making it essential to reduce cement consumption [4,5,6,7]. However, as cement consumption increases, backfilling costs inevitably rise; conversely, reducing cement content leads to a decline in the strength of the backfill material [8,9]. As a result, reducing cement costs has become a key focus of research and exploration for many scholars [10,11]. To reduce cement consumption, filling cementitious materials can be prepared using refining slag, ground granulated blast furnace slag, steel slag, and desulfurized gypsum as substitutes for cement [12]. Additionally, researching high-performance and low-cost binder materials offers another promising approach to lowering cement-related expenses [13]. The addition of fiber materials also provides a cost-effective solution without compromising environmental sustainability [14,15]. Widely used artificial and natural fibers include polypropylene (PP) and rice straw (RS) fibers [16,17].

Since the discovery of PP, it has been widely used across various fields due to its excellent mechanical properties, heat resistance, and other beneficial characteristics [18,19]. Cai et al. [20] used a mixture of PP fiber and lime for ground improvement, finding that the combination significantly enhanced the engineering properties of the treated soil. Li et al. [21] investigated the crack behavior of PP fiber-reinforced geopolymer mortar, identifying the optimal fiber content and length. Aisheh et al. [22] studied the impact of PP fibers on the mechanical properties of ultra-high-performance geopolymer concrete composites, demonstrating improvements. Yi et al. [23] conducted research on the use of PP fiber-reinforced cemented paste backfill to enhance the stability of underground mine backfills and reduce cement consumption. While PP fibers have been demonstrated to strengthen cemented paste backfill, no studies have yet investigated their reinforcing effects on TWSWB.

RS, the most common agricultural waste in China, plays an important role in certain industries, especially the construction sector [24,25,26]. As a waste product, RS is most commonly disposed of through incineration, which contributes to air pollution [27]. However, other disposal methods, such as using RS in paper production, as livestock feed, and for other purposes, are also prevalent [28]. AI et al. [29] found that adding appropriate amounts of RS to asphalt mixtures enhanced their physical properties and strain resistance. In addition, the presence of RS fiber has an effect on the hydration of cement-based composites, thereby affecting their strength after curing [30,31]. Wang et al. [32] found that alkalized RS improved the mechanical properties of cemented tailings backfill, reducing cement usage. For TWSMB, which requires a large amount of cement, the addition of RS fibers is significant for improving its strength.

In summary, PP fibers exhibit excellent mechanical properties and heat resistance, improving the strength of concrete, while RS fibers can also influence the strength of cured concrete. Both types of fibers are widely used in construction and road engineering. While some studies have explored the significance of these fibers in mine backfill, limited research has been conducted on their application in TWSMB [33,34,35]. Therefore, this study investigates the effects of PP and RS fibers on the strength of TWSMB. Three experimental groups were tested: unreinforced, PP fiber-reinforced, and RS fiber-reinforced. An orthogonal experiment was designed, and the fluidity of each group was tested. The mechanisms of the two types of fibers in TWSMB were studied through macroscopic observation and scanning electron microscopy (SEM).

## 2. Materials and Methods

### 2.1. Raw Materials and Specimen Preparation

#### 2.1.1. Graded Tailings, Wasted Stone, Cementitious Material, and Water

**Figure 1:**
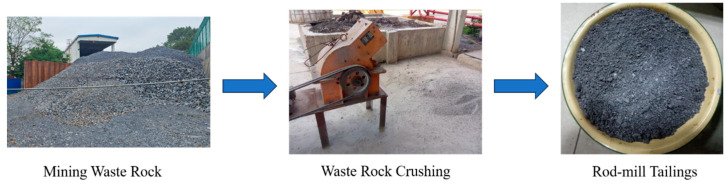
Rod-mill tailings preparation process.

**Figure 2:**
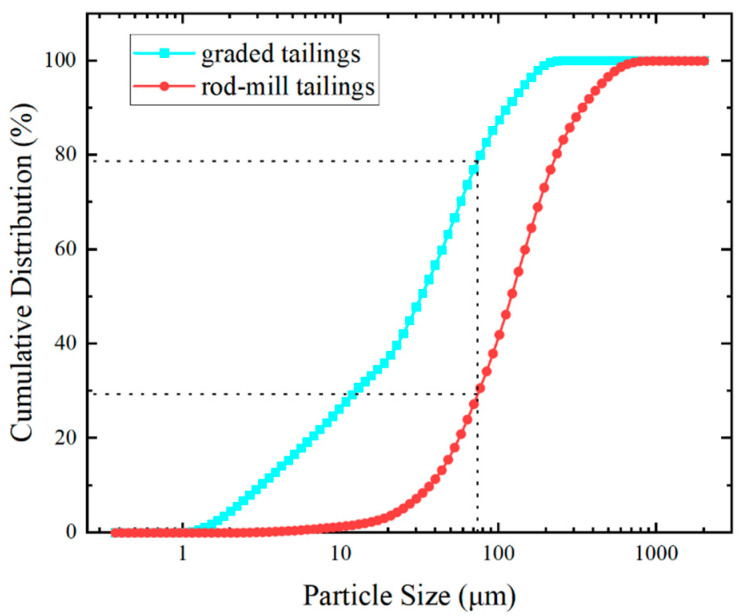
Cumulative particle size distribution of graded tailings and rod-mill tailings.

In this research, graded tailings from a lead–zinc mine were used as the primary fill aggregate. Additionally, mine waste rock was incorporated as a filling material to assess its impact on the strength of the backfill. To ensure the slurry could be transported to the stope, the waste rock was ground into rod-mill tailings. The preparation flow diagram is shown in Figure 1, and the cumulative particle size distribution of graded tailings and rod-mill tailings is presented in Figure 2. The particle size of graded tailings reaches 74 μm with a cumulative distribution of nearly 80%, whereas for rod-mill tailings, the cumulative distribution is below 30% at the same particle size. Compared to graded tailings, rod-mill tailings have a larger particle size, and their addition enhances the stability and strength of the backfill. The measured physical parameters of graded tailings and rod-mill tailings are shown in Table 1. It can be observed that the water permeability of rod-mill tailings is better. Table 2 lists the chemical composition measurements of graded tailings and rod-mill tailings measured by an X-ray fluorescence spectrometer (XRF). The primary oxides in both graded and rod-mill tailings are CaO, SiO_2_, Al_2_O_3_, Fe_2_O_3_, and MgO, which together account for over 85% of the total composition. Specifically, the SiO_2_ content in graded tailings is 16.80%, while its sulfur (S) content is 0.91%. In rod-mill tailings, the SiO_2_ content is 24.60%, while the S content is much higher at 10.70%. Although the SiO_2_ content in rod-mill tailings is greater than in graded tailings, the significantly higher S content in rod-mill tailings can negatively impact the backfill, reducing its strength [36].

To ensure the TWSMB meets the required strength for mining operations, it is essential to incorporate cementitious materials to produce a slurry that satisfies the necessary standards. In this study, all specimens were prepared using Portland cement.

#### 2.1.2. Polypropylene Fiber and Straw Fiber

PP is a versatile thermoplastic material with many mechanical advantages. With a density of 0.90–0.92 g/cm^3^, PP fiber is the lightest among all chemical fibers. It also boasts excellent properties such as high strength, good elasticity, wear resistance, and corrosion resistance [37]. Typically, the length of PP fiber is between 15~50 mm, but in this study, fibers ranging from 3~12 mm were used to reduce material cost while enhancing the strength of the test specimens. The PP fibers used in the experiment have an average diameter of 19 μm, a density of 910 kg/m^3^, and a COV value of approximately 7–8%, exhibit excellent toughness. These PP fibers have a fracture strength greater than 350 MPa and an elastic modulus greater than 3.5 GPa, which enables the improvement of the mechanical properties of TWSMB when mixed with PP fibers. These PP fibers were provided by the manufacturer and have undergone rigorous testing. Additionally, the PP fiber content was limited to 0.05–0.2% of the total mass of tailings and cement to further mitigate the high costs associated with fiber addition. The study primarily focuses on investigating the impact of fiber concentration and length on the strength of TWSMB.

**Figure 3:**
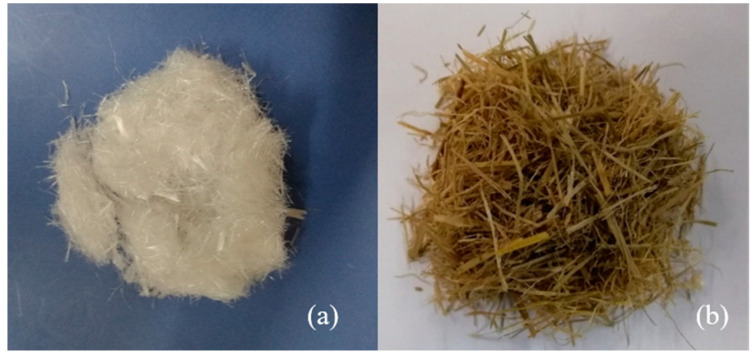
PP fiber and RS fiber: (**a**) PP fiber, (**b**) RS fiber.

Rice is one of the most common staple crops in China, and the straw waste left after harvesting, known as RS, is a readily available material for this study. Compositionally, RS fiber is a natural fiber rich in cellulose, hemicellulose, lignin, and ash, and it possesses excellent moisture absorption properties [38]. Its density typically ranges from 90 to 120 kg/m^3^, with a tensile strength of 45–50 MPa, an elastic modulus of around 20 GPa, and a COV value of 10–15%, all of which were tested and provided by the supplier. In this study, the RS fiber was dried and then cut into filamentous straw fibers with a width not exceeding 3 mm and a length of 5–50 mm. Unlike the PP fiber experiment, the RS fiber content in the specimens ranged from 0.5 to 1.5 g/m^3^, with the proportion of rod-mill tailings in solid mass concentration between 0 and 1.0 wt.%. Figure 3 shows the PP fibers and RS fibers utilized in this study.

#### 2.1.3. Details of the Specimens Preparation

In this study, three types of specimens were prepared: unreinforced, PP fiber-reinforced, and RS fiber-reinforced. Five influencing factors were controlled as variables: cement–sand ratio, solid mass concentration, fiber content, fiber length, and the proportion of rod-mill tailings in solid mass concentration. Different cement–sand ratios and solid mass concentrations were tested to improve the strength of TWSMB, as these factors significantly influence its mechanical properties. Unreinforced, PP fiber-reinforced and RS fiber-reinforced experimental groups required the preparation of specific specimens based on these variable conditions. Moreover, unconfined compressive strength (UCS) tests were required for specimens after 3, 7, and 28 days of curing (hereinafter referred to as 3 d, 7 d, and 28 d).

**Figure 4:**
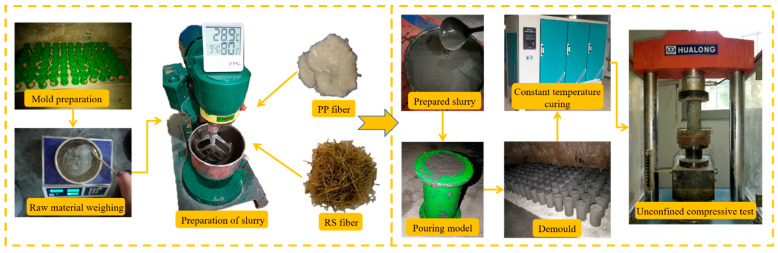
Specimens preparation process.

The fiber specimen production process was conducted as follows: First, the cement, tailings, and rod-mill tailings were initially mixed for 5 min in a laboratory cement mortar mixer, and fibers were added after the beginning of mixing to avoid the floating of fibers. Water was then slowly introduced into the mixer, and the mixture was stirred for an additional 5 min to ensure thorough blending. The mixture was then poured into plastic molds with a diameter of 5 cm and a height of 10 cm. After demolding, the prepared specimens were placed in a standard curing box at 22 ± 1 °C and 90% RH for 3 d, 7 d, and 28 d. The specific specimens preparation flow chart is shown in Figure 4.

### 2.2. Experimental Methods

#### 2.2.1. Orthogonal Tests

In this study, the orthogonal experiment method was employed to improve experimental efficiency while preparing a large number of specimens using the control variable method. This approach, which is high-efficiency, rapid, and economical, is particularly suitable for multi-factor and multi-level experiments as it significantly reduces the workload. Table 1 outlines the factor design for the unreinforced, PP fiber-reinforced, and RS fiber-reinforced test groups. The orthogonal test was designed with five factors including cement–sand ratio (A), solid mass concentration (B), fiber content (C), fiber length (D), and the proportion of rod-mill tailings in solid mass concentration (E). Each factor has four levels as follows: cement–sand ratios of 1/8, 1/10, 1/12, and 1/14; solid mass concentrations of 73, 75, 77, and 79 wt.%; fiber concentrations of 0.5, 1.0, 1.5, and 2.0 kg/m^3^; and the proportion of rod-mill tailings in solid mass concentration of 0, 0.5, 1.5, and 2.0 wt.%. Notably, the optimal lengths of PP fiber and RS fiber differ in enhancing the strength of TWSMB. To maintain slurry fluidity, the four length levels for PP fiber are set at 3, 6, 9, and 12 mm, while the three length levels for RS fiber are set at 5–10, 10–30, and 40–50 mm.

The three experimental groups, unreinforced, PP fiber-reinforced, and RS fiber-reinforced, also serve as control groups for comparative analysis. Under identical conditions, comparing the UCS results of the unreinforced group with those of the PP and RS fiber-reinforced groups allows for evaluation of the impact of fiber addition on TWSMB strength. These experimental groups are labeled as T (unreinforced), P (PP fiber-reinforced), and R (RS fiber-reinforced), with the numbers following the letters representing the specimen numbers under different mix ratio conditions.

#### 2.2.2. Diffusivity Test of Backfill

**Figure 5:**
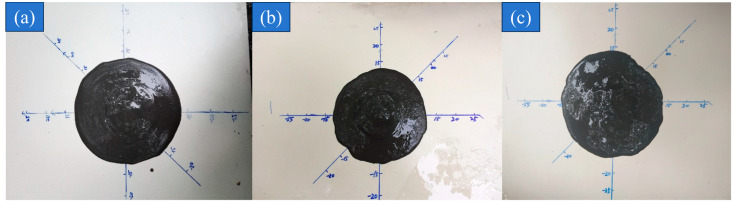
The diffusivity of slurry: (**a**) unreinforced, (**b**) PP fiber-reinforced, (**c**) RS fiber-reinforced.

Diffusivity serves as a comprehensive indicator of filling slurry flow performance, directly reflecting its flow behavior [39]. Figure 5 shows the diffusivity of unreinforced, PP fiber-reinforced, and RS fiber-reinforced slurry (P.O 42.5R) and tap water. In this study, the diffusivity of the freshly prepared TWSMB slurry mixture was determined by measuring the diameter of its spread using a small diffusion cylinder. The small diffusion cylinder is a smooth surface cylinder with an upper and lower diameter and height of 8 cm. At first, the diffusion cylinder should be cleaned, placed on a graduated board, filled with the slurry, and scraped flat. Then, quickly lift the diffusion cylinder vertically, allowing the slurry to spread into a circular shape on the board. The diffusivity is calculated as the average of two perpendicular diameter measurements of the resulting circle. Based on long-term operational experience in the lead–zinc mine, a slurry diffusivity of at least 20 cm is required for effective flow transportation.

#### 2.2.3. Unconfined Compressive Strength Tests

The UCS is an important mechanical property index used to evaluate the strength performance of the specimens. The curing time was divided into three periods: 3 d, 7 d, and 28 d. During each period, three specimens were tested repeatedly under the same conditions to minimize experimental errors. This approach helps effectively monitor the strength development of the specimens before they fully reach 100% strength. For each curing time, the UCS of the prepared TWSMB specimen was tested using a computer-controlled automatic pressure testing machine, with the average UCS of the replicated specimens taken as the final result.

#### 2.2.4. Scanning Electron Microscopy

SEM is an effective method for observing the microstructure of filler slurries, which is widely used to analyze the microstructure of concrete and its components, and is recommended by ASTM as a research standard to study concrete [40]. Cement exists as a cementitious material in TWSMB, and SEM observation of the microstructure of TWSMB provides valuable reference information. In this study, a field-emission electron microscope system (QUANTA FEG 250, FEI, Hillsboro, OR, USA) was employed to examine PP fiber- and RS fiber-reinforced specimens and observe the microstructure of TWSMB. Images were captured at various magnifications to analyze the structure. Before testing, the specimens must be processed into thin sections and coated with a conductive layer using a high vacuum coating instrument (EM SCD 500, LEICA, Wetzlar, Germany) to ensure electrical conductivity.

## 3. Results and Discussion

### 3.1. Liquidity and UCS Results

Before examining the mechanical properties of fiber-reinforced TWSMB, the diffusivities of unreinforced, PP fiber-reinforced, and RS fiber-reinforced slurries were measured. The diffusivity values were found to be 22.850–33.475 cm, 20.750–29.613 cm, and 23.350–30.667 cm, respectively. These results indicate that the diffusivity of the slurries in all three groups was similar. Specifically, the diffusivity of the PP fiber-reinforced slurry showed a slight decrease, while the diffusivity of the RS fiber-reinforced slurry slightly increased. The result suggests that the addition of PP and RS fibers to the TWSMB slurry does not significantly reduce its fluidity. Figure 5a–c show the experimental results for the diffusivity of the unreinforced, PP fiber-reinforced, and RS fiber-reinforced slurries.

### 3.2. The Analysis of UCS

**Figure 6:**
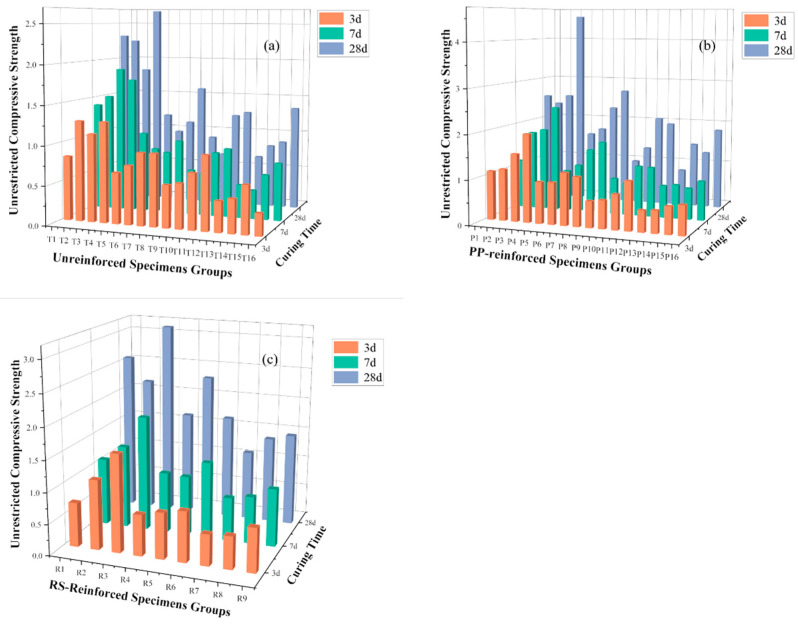
The UCS of specimens: (**a**) Unreinforced specimens, (**b**) PP fiber-reinforced specimens, (**c**) RS fiber-reinforced specimens.

In Figure 6, panels (a–c) show the UCS of unreinforced, PP fiber-reinforced, and RS fiber-reinforced specimens under different cement–sand ratios, solid mass concentrations, fiber contents, fiber lengths, and proportions of rod-mill tailings in solid mass concentration. The detailed orthogonal mix design can be found in Table 3. It can be observed that the UCS of PP fiber-reinforced and RS fiber-reinforced specimens is generally higher than that of unreinforced specimens, indicating that fiber reinforcement improves the strength of TWSMB. At 3 d, fewer unreinforced specimens reached a UCS of more than 1 MPa compared to those reinforced with PP or RS fibers. The maximum UCS values of PP fiber-reinforced and RS fiber-reinforced TWSMB specimens at 3 d, 7 d, and 28 d were 1.98, 2.42, and 4.55 MPa, and 1.57, 1.86, and 3.13 MPa, respectively. In contrast, the maximum UCS values of unreinforced TWSMB specimens at 3 d, 7 d and 28 d were 1.28, 1.89, and 2.67 MPa, respectively. Generally, the strength of the unreinforced specimens was lower than that of the fiber-reinforced specimens, with the exception of the RS fiber-reinforced specimens at 7 d, which showed lower strength. The strength of the fiber-reinforced TWSMB specimens improved overall, with the maximum UCS increase for RS fiber being 70.11%, while the maximum UCS increase for PP fiber reached 108.11%. This improvement can be attributed to the reinforcement provided by PP and RS fibers. Both fiber types are relatively flexible, ensuring better contact between particles, which makes the TWSMB denser and results in higher strength and better integrity to resist external pressure.

Additionally, as shown in Figure 6, the strength of the specimens increases with higher cement–sand ratios and solid mass concentrations. The addition of more cement enhances the bonding effect and strength of the filling slurry. The strength of TWSMB increases with longer curing times, as indicated by the graph. The effect of PP fiber on the strength of the backfill is more pronounced in the early and later stages than in the middle stage. In contrast, the effect of RS fiber on strength in the medium term may be minimal or even negative. However, after 28 d, both PP and RS fibers contributed to higher strength, with the effect of RS fiber becoming more evident in the later curing stages.

### 3.3. Macrostructural Failure

**Figure 7:**
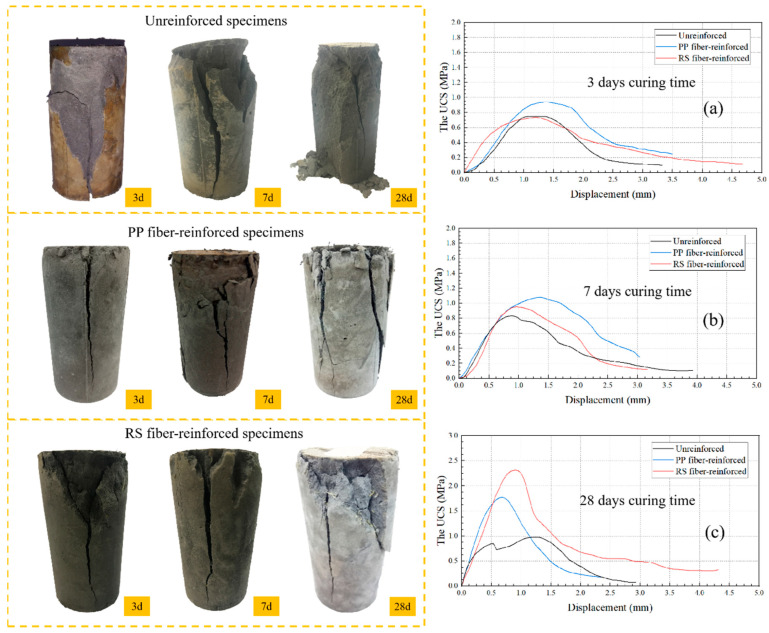
Macroscopic failure of different specimens at different curing times; figure (**a**–**c**) show the stress–strain curves of different specimens at 3 d, 7 d, and 28 d, respectively.

Macroscopic failure analysis provides a clear and direct observation of the failure patterns of TWSMB. The automatic pressure testing machine applied a constant displacement rate of 0.2 mm/min to load the specimens, recording stress and displacement data every second in an Excel file until the specimen was destroyed. Analyze the stress–strain curves of T6, P6, and R5 specimens in conjunction with their macroscopic failure. As shown in Figure 7, the failure modes of unreinforced, PP fiber-reinforced, and RS fiber-reinforced TWSMB specimens were different. Compared to the unreinforced specimens, the fiber-reinforced specimens exhibit significantly smaller cracks, while the unreinforced specimens even show large chunks detaching from the matrix. This can be attributed to the pulling action of the fibers, which helps to restrain the deformation of the matrix under load, resulting in less crack propagation and more uniform stress distribution within the material. The stress–strain curves in the figure show that both PP fiber-reinforced and RS fiber-reinforced specimens achieve higher ultimate strengths than the unreinforced specimens, along with more pronounced displacement at ultimate strength, which indicate a superior ability to resist displacement deformation. Furthermore, this suggests that fiber reinforcement not only enhances the strength of the backfill but also increases its toughness. The UCS values achieved at 28 d for PP fiber- and RS fiber-reinforced specimens are 1.76 MPa and 2.33 MPa, respectively, which are 0.79 MPa and 1.36 MPa higher than those of the unreinforced specimens. After failure, compared to the unreinforced specimens, the residual load-bearing capacity of the PP fiber-reinforced and RS fiber-reinforced specimens is greater, enabling them to sustain higher strength at certain deformation levels. Even after exceeding the ultimate compressive strength, the fiber-reinforced TWSMB specimens maintain their integrity and residual strength. This is because the fibers serve to bridge cracks and prevent crack propagation. After being mixed with TWSMB, the fibers adhere tightly to the materials within the specimen, helping to prevent premature failure and enhancing both its strength and ductility. When failure does occur, the fibers pull on the specimen, preventing it from completely breaking apart.

### 3.4. Effect of Both Fiber Content and Length

#### 3.4.1. Orthogonal Range Analysis

The most commonly used method, orthogonal range analysis, was used in the analysis of orthogonal test results in this study. It can be used to determine the degree of influence of different factors, which can be calculated by Equation (1):

(1) $$R_{j}=\max\left(\bar{K_{j1}},\bar{K_{j2}},\cdots ,\bar{K_{\mathrm{jm}}}\right)\;-\;\min\left(\bar{K_{j1}},\bar{K_{j2}},\cdots ,\bar{K_{\mathrm{jm}}}\right)$$

where $R_{j}$ is the range of the test index as the level of column j changes, and the greater the value of $R_{j}$ is, the greater the impact of the factor on the test index; $\bar{K_{\mathrm{jm}}}$ represents the average value of the level of column j.

In this study, the influence of five factors, namely, cement–sand ratio (A), solid mass concentration (B), fiber content (C), fiber length (D), and the proportion of rod-mill tailings in solid mass concentration (E), on the UCS of the backfill was judged by the size of the orthogonal range analysis value $R_{j}$.

#### 3.4.2. The Influence of Factors A, B, and E

**Figure 8:**
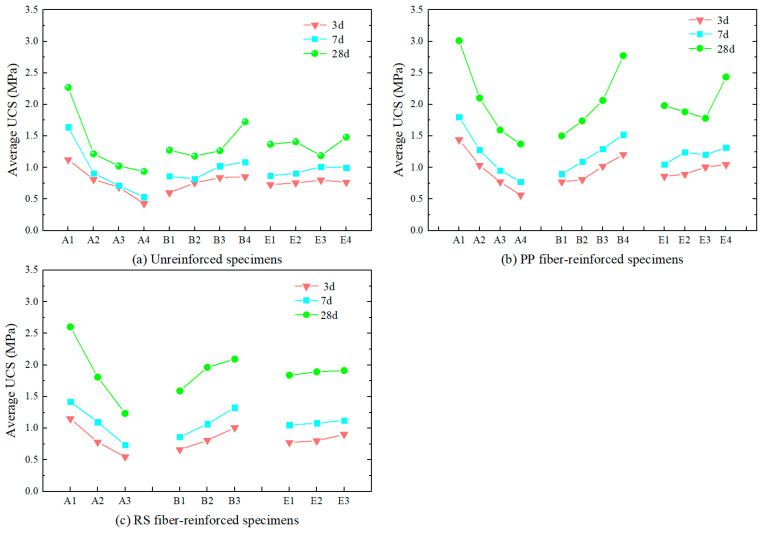
A, B, and E factors influence the results of orthogonal test of unreinforced, PP fiber-reinforced, and RS fiber-reinforced specimens.

As shown in Figure 8, the variation in the average UCS values for the unreinforced, PP fiber-reinforced, and RS fiber-reinforced specimens at different levels of factors A, B, and E can be clearly observed. It is clear that the influence of factors A and B is significantly greater than that of factor E. Both factors A and B have a significant impact on the TWSMB strength, with the cement–sand ratio having the most pronounced effect. The order of influence on UCS is A > B > E, which is also reflected in the PP fiber-reinforced and RS fiber-reinforced specimen groups. Furthermore, it can be seen that the UCS values of the PP fiber-reinforced and RS fiber-reinforced specimens are generally higher than those of the unreinforced specimens, indicating that the addition of fibers effectively enhances the TWSMB strength. Based on the strength results, while fiber parameters are not the most influential factors in determining TWSMB strength, they play an important role in improving the UCS of the backfill.

#### 3.4.3. Fiber Content and Fiber Length

**Figure 9:**
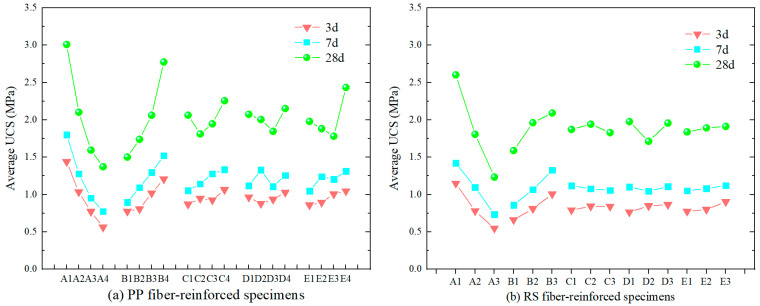
Orthogonal test results of PP fiber-reinforced and RS fiber-reinforced specimens influenced by five factors.

In Figure 9, the analysis of the PP fiber-reinforced specimen group (Figure 9a) and the RS fiber-reinforced specimen group (Figure 9b) reveals the differences between the factors influencing UCS. The influence of the factors on UCS in the PP fiber-reinforced and RS fiber-reinforced specimens is found to follow the following order: A > B > E > C > D and A > B > E > D > C, respectively. Notably, the differences in fiber concentration and fiber length between PP fibers and RS fibers may be attributed to the distinct mechanisms by which each fiber type influences the TWSMB.

The strength of PP fiber-reinforced specimens exhibited a steady increase for the first 3 and 7 days of curing, while the strength of specimens cured for 28 days initially decreased before increasing. From the strength enhancement trend, it is evident that the optimal fiber content for PP fibers is 1.5%, as the UCS of the specimen reached 2.257 MPa after 28 d. In contrast, the strength of RS fiber-reinforced specimens increased steadily at 3 d, decreased steadily at 7 d, and initially increased before decreasing at 28 d. Based on the trend of strength enhancement, the optimal fiber content for RS fibers is 1%, with the UCS of the specimen reaching 1.943 MPa after 28 d.

Regarding fiber length, the UCS of PP fiber-reinforced specimens showed that as the fiber length increased, the strength of the specimens cured for 3 days and 28 days initially decreased and then increased. However, for specimens cured for 7 days, the strength increased and then decreased. Based on the experimental data, PP fibers with a length of 6 mm provided the best reinforcement effect. For RS fiber-reinforced specimens, the strength of the 3-days-cured specimens increased with the extension of fiber length. However, for the specimens cured for 7 and 28 days, the strength initially decreased and then increased, with the maximum UCS reaching 1.977 MPa at 28 days when the fiber length was between 5–10 mm. Therefore, RS fibers with a length of 5–10 mm provided the best strength enhancement.

In summary, the optimal configuration for PP fibers is a concentration of 1.5% with a fiber length of 6 mm, while for RS fibers, a concentration of 1.0% with a fiber length of 5–10 mm produced the best performance. The findings indicate that unrestricted increasing fiber concentration and length does not maximize the strength of TWSMB, as each fiber type has its own optimal parameters. The addition of fibers enhances the structural interaction among tailings particles, thereby increasing TWSMB strength, but it also introduces weak planes within the structure. Higher fiber concentrations and lengths tend to create more weak points, potentially reducing TWSMB strength. Among all specimens tested, the UCS of PP fiber-reinforced specimens consistently exceeded that of RS fiber-reinforced specimens, with PP fiber demonstrating a greater strength increase than RS fiber.

### 3.5. SEM Tests

**Figure 10:**
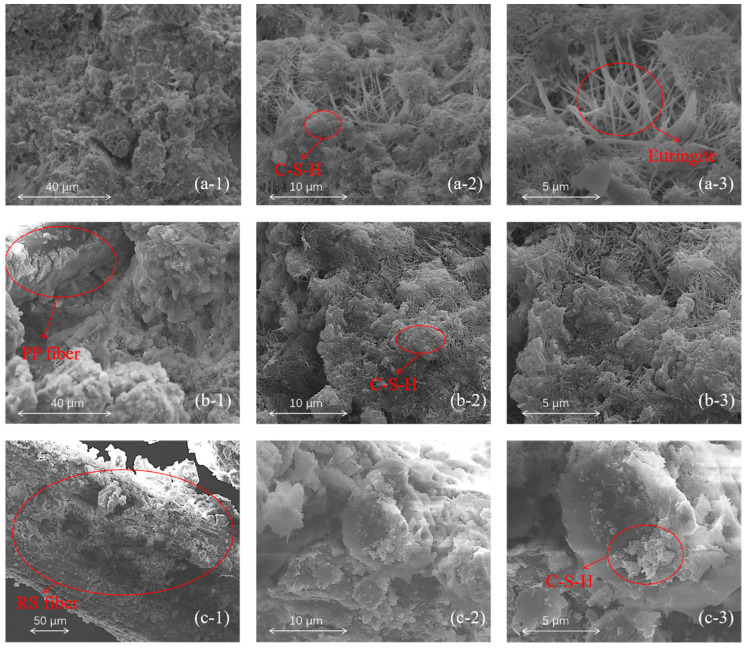
SEM images of specimens under different magnifications: (**a-1**, **a-2** and **a-3**) are images of unreinforced specimens magnified 3000×, 10,000×, and 20,000×, respectively; (**b-1**, **b-2** and **b-3**) are images of PP fiber-reinforced specimens magnified 3000×, 10,000×, and 20,000×, respectively; (**c-1**, **c-2** and **c-3**) are images of RS fiber-reinforced specimens magnified 1000×, 5000×, and 10,000×, respectively.

SEM analysis provides a highly informative method for exploring the relationship between UCS and the microstructure of TWSMB specimens. Figure 10 displays the microstructures of unreinforced (row “a”), PP fiber-reinforced (row “b”), and RS fiber-reinforced (row “c”) TWSMB specimens after 28 d, with images at varying magnifications in each row. In row “a”, the microstructure of the unreinforced TWSMB shows that intermediate tailings and cement generate a large amount of hydrated calcium silicate (C-S-H) and ettringite after 28 days of hydration reaction [33,41]. Images (b-1) (40 μm) and (c-1) (50 μm) in rows “b” and “c” clearly illustrate the interactions of these hydration products with PP and RS fibers.

In the PP fiber-reinforced specimen (row “b”), C-S-H and ettringite adhere to the PP fibers, embedding them within the particle-binder matrix. This interaction maximizes the tensile strength of the PP fibers, thereby enhancing the UCS of the specimen. Higher-magnification images reveal that the PP fiber-reinforced specimen has a denser structure than the unreinforced specimen, contributing to its superior compressive strength. This structural improvement is why PP fiber-reinforced TWSMB exhibits the highest compressive strength among the three specimen groups.

The microstructure of RS fiber-reinforced specimens (row “c”) shows distinct differences from the PP fiber-reinforced specimens. Specifically, upon magnification, it is observed that larger C-S-H particles are primarily wrapped around the surface of the RS fibers, with the fiber surface almost entirely covered by hydration products. A physical skeleton structure is formed between the RS fibers and the hydration products, as many hydration products are trapped on the surface of the RS fibers due to their strong interaction with the hydration products, which significantly enhances the ability of TWSMB to withstand greater pressure [34].

## 4. Superiority and Limitations

Based on the experimental results, the addition of fibers can increase the strength of TWSMB specimens, providing a new option for reducing the use of cement in TWSMB. Reducing cement usage also helps lower CO_2_ emissions during the filling process and reduces the cost of cement. In addition, the incorporation of fibers helps to maintain the integrity of the fill and enhances its residual strength.

Currently, the industrial application of fiber-reinforced fill has not been widely adopted. It is important to note that the addition of fibers to the fill slurry may have an impact on the mixing process. However, this study only involved the reinforcement effects of PP fibers and RS fibers on TWSMB, and the reinforcement effects of other artificial and natural fibers still require further investigation. Additionally, the optimal fiber reinforcement ratio may be constrained by the composition of the tailings from this particular mine, and the best fiber reinforcement ratio may vary for different mines.

Previous studies have shown that while these fibers can enhance short-term strength and provide a bridging effect, their effectiveness may decrease over time due to potential degradation and weakening. In particular, RS fibers degrade more quickly than PP fibers, and their strength may be compromised under prolonged degradation conditions [42,43]. We will further investigate their long-term strength performance in future studies to confirm these findings.

## 5. Conclusions

Three types of experiments with different fiber conditions were carried out to study the influence of different factors on the strength of TWSMB. Orthogonal experiments were designed for unreinforced, PP fiber-reinforced, and RS fiber-reinforced specimens, respectively. The experimental specimens were tested for unconfined compressive performance and SEM analysis. The following are the conclusions of the study:

The diffusivity of unreinforced, PP fiber-reinforced, and RS fiber-reinforced fillers was 22.850~33.475 cm, 20.750~29.613 cm, and 23.350~30.667 cm, respectively. The addition of both types of fibers had minimal impact on the liquidity of TWSMB.

The UCS results indicated that the cement–sand ratio is the most significant factor affecting TWSMB strength, followed by the solid mass concentration. The proportion of rod-mill tailings in solid mass concentration had the least influence. The addition of PP or RS fibers improves the strength of TWSMB, with a larger impact than the proportion of rod-mill tailings in solid mass concentration. However, PP fibers demonstrated a slightly greater strength-enhancing effect than RS fibers.

The experimental results show that the optimal fiber configuration is 1.5% mass concentration and 6 mm length for PP fibers, and 1.0% mass concentration with a fiber length of 5–10 mm for RS fibers. Adding the appropriate proportion and length of PP or RS fibers to TWSMB can significantly reduce the cement content, thereby lowering the backfill cost.

Macroscopic damage analysis revealed that fibers have a crack-bridging and reinforcing effect on TWSMB specimens. The inclusion of fibers helps prevent premature failure of TWSMB, enhancing its strength and ductility.

SEM observations revealed that C-S-H and ettringite adhered to the PP fibers, encapsulating them and fixing them in the particle-binder matrix, forming a dense structure. SEM analysis of RS fibers showed that larger C-S-H was mainly wrapped on the surface of the RS fibers. Their surface was almost completely covered by hydration products, forming a physical skeleton structure.

## Figures and Tables

**Table 1:** Physical parameters of graded tailings and rod-mill tailings.

Physical Parameters	Graded Tailings	Rod-Mill Tailings	Unit
Wet density	2.16	1.93	g/cm^3^
Dry density	1.93	1.86	g/cm^3^
True density	3.18	2.80	g/cm^3^
Porosity	39.31	33.57	%
Osmotic coefficient	8.70 × 10^−4^	3.80 × 10^−3^	cm/s

**Table 2:** Chemical composition of graded tailings and rod-mill tailings.

Graded tailings	Chemical composition	Al_2_O_3_	SiO_2_	MnO	Fe_2_O_3_	MgO	CaO	K_2_O	TiO_2_	P_2_O_5_	S	Cu
	Amount (%)	5.69	16.80	0.19	4.57	3.51	65.2	1.49	0.37	0.05	0.910	0.010
	Chemical composition	Sr	Cr	Ni	Zn	Pb	Ba	Cl	F	Zr	As	Rb
	Amount (%)	0.032	0.098	0.008	0.732	0.228	/	0.018	/	0.008	/	0.006
Rod-mill tailings	Chemical composition	Al_2_O_3_	SiO_2_	MnO	Fe_2_O_3_	MgO	CaO	K_2_O	TiO_2_	P_2_O_5_	S	Cu
	Amount (%)	5.21	24.60	0.28	22.50	1.63	32.0	1.18	0.24	0.06	10.70	0.013
	Chemical composition	Sr	Cr	Ni	Zn	Pb	Ba	Cl	F	Zr	As	Rb
	Amount (%)	0.022	0.076	0.009	0.448	0.47	0.061	0.019	0.337	0.007	0.084	0.004

**Table 3:** Orthogonal design table of unreinforced, PP fiber-reinforced, RS fiber-reinforced test groups.

Unreinforced	Specimen Group	T1	T2	T3	T4	T5	T6	T7	T8	T9	T10	T11	T12	T13	T14	T15	T16
	A	1:8	1:8	1:8	1:8	1:10	1:10	1:10	1:10	1:12	1:12	1:12	1:12	1:14	1:14	1:14	1:14
	B ^1^ (wt.%)	73	75	77	79	73	75	77	79	73	75	77	79	73	75	77	79
	E ^3^ (wt.%)	0	0.05	0.1	0.15	0.15	0.1	0.05	0	0.05	0	0.15	0.1	0.1	0.15	0	0
PP fiber-reinforced	Specimen group	P1	P2	P3	P4	P5	P6	P7	P8	P9	P10	P11	P12	P13	P14	P15	P16
	A	1:8	1:8	1:8	1:8	1:10	1:10	1:10	1:10	1:12	1:12	1:12	1:12	1:14	1:14	1:14	1:14
	B (wt.%)	73	75	77	79	73	75	77	79	73	75	77	79	73	75	77	79
	C ^2^ (g/m^3^)	0.5	1	1.5	2	1	0.5	2	1.5	1.5	2	0.5	1	2	1.5	1	0.5
	D (mm)	3	6	9	12	9	12	3	6	12	9	6	3	6	3	12	9
	E (wt.%)	0	0.05	0.1	0.15	0.15	0.1	0.05	0	0.05	0	0.15	0.1	0.1	0.15	0	0.05
RS fiber-reinforced	Specimen group	R1		R2	R3		R4		R5	R6		R7		R8		R9	
	A	1:8		1:8	1:8		1:10		1:10	1:10		1:12		1:12		1:12	
	B (wt.%)	73		75	77		73		75	77		73		75		77	
	C (g/m^3^)	0.5		1	1.5		0.5		1	1.5		1		1.5		0.5	
	D (mm)	5–10		10–30	40–50		10–30		40–50	5–10		5–10		10–30		40–50	
	E (wt.%)	0		0.05	0.1		0.05		0.1	0		0.1		0		0.05	

^1^ Solid mass includes tailing, cement, fiber, and rod-mill tailing; ^2^ the content of fiber added to the slurry; ^3^ ratio of rod-mill tailings to solid aggregate.

## Data Availability

The original contributions presented in the study are included in the article, further inquiries can be directed to the corresponding author.
